# Insights into the Prognostic Efficacy of the Geriatric Nutritional Risk Index for Nasopharyngeal Carcinoma in the Era of Volumetric Modulated Arc Therapy: A Nomogram for Predicting Long-Term Survival Outcomes

**DOI:** 10.3390/curroncol32070372

**Published:** 2025-06-26

**Authors:** Xiang Lin, Wei Wang, Jianming Ding, Zhaodong Fei, Chuanben Chen

**Affiliations:** Department of Radiation Oncology, Clinical Oncology School of Fujian Medical University, Fujian Cancer Hospital, 91 Maluding, Fuma Road, Fuzhou 350014, China; linxiang@fjzlhospital.com (X.L.); dr_wangwei@sina.com (W.W.); djm_1991@fjmu.edu.com (J.D.); feizhaodong@fjmu.edu.cn (Z.F.)

**Keywords:** geriatric nutritional risk index, prognosis, nasopharyngeal carcinoma, nomogram, volumetric modulated arc therapy

## Abstract

Background: The geriatric nutritional risk index (GNRI), a composite metric of serum albumin and body weight, has emerged as a prognostic tool in various cancers. However, its relevance in nasopharyngeal carcinoma (NPC) patients treated with volumetric modulated arc therapy (VMAT) remains unexplored. The aim of this study was to assess the effect of the GNRI in the prediction of the prognosis of nasopharyngeal carcinoma in the era of VMAT. Methods: This retrospective study analyzed 498 newly diagnosed, non-metastatic NPC patients treated with VMAT between 2010 and 2011. The GNRI was calculated using serum albumin and body weight ratios, with receiver operating characteristic (ROC) curve analysis determining its optimal prognostic cutoff. Patients were stratified into training (70%) and validation (30%) cohorts. Cox regression identified independent prognostic factors, which were integrated into a nomogram predicting 3- and 5-year overall survival (OS). Model performance was assessed via the concordance index (C-index), calibration curves, and decision curve analysis (DCA). Results: In the study, 348 patients were included in the training cohort and 150 patients were included in the validation cohort according to a ratio of 7:3. The median follow-up was 68 months, with 5-year OS rates of 79.3%. A GNRI > 102 independently predicted improved survival (HR = 0.64; *p* = 0.044), alongside tumor volume, age, and N-stage. The nomogram demonstrated strong discrimination (C-index: 0.757–0.762 for training; 0.737–0.744 for validation) and calibration, aligning closely with observed survival. DCA confirmed superior clinical utility over default strategies. NPC patients treated with VMAT with a high GNRI, female sex, and a lower N-stage exhibited significantly better OS (*p* < 0.05). Conclusions: The GNRI is a robust prognostic marker for NPC patients receiving VMAT, reflecting the interplay of nutrition, inflammation, and treatment response. The validated nomogram provides a practical tool for individualized risk stratification, enhancing clinical decision-making in the era of precision radiotherapy.

## Simple Summary

Nasopharyngeal carcinoma, a cancer common in Asia, is often treated with advanced radiotherapy called volumetric modulated arc therapy. Good nutrition is vital for cancer patients, but its role in nasopharyngeal carcinoma survival with modern volumetric modulated arc therapy treatment is unclear. This study analyzed 498 nasopharyngeal carcinoma patients and found that the geriatric nutritional risk index, a simple score based on blood protein and weight, strongly predicted long-term survival. Patients with higher geriatric nutritional risk index (>102) lived longer. Researchers combined the geriatric nutritional risk index with tumor size, age, and cancer stage to create an easy-to-use prediction tool. This tool helps doctors estimate survival chances at 3 and 5 years after treatment, allowing personalized care plans. The findings highlight the importance of nutrition in nasopharyngeal carcinoma outcomes and support tailored treatment approaches in the era of precision radiotherapy.

## 1. Introduction

Nasopharyngeal carcinoma (NPC) represents a distinct epithelial malignancy originating from the nasopharyngeal mucosal lining, characterized by a striking geographical predilection with endemic incidence rates concentrated in Southern China and Southeast Asia, particularly in China’s Guangdong and Fujian provinces [1,2]. Global epidemiological data from the International Agency for Research on Cancer indicated that nasopharyngeal carcinoma accounted for approximately 133,000 incident cases and 80,000 attributable deaths worldwide in 2020 [3]. Radiotherapy serves as the therapeutic cornerstone for non-metastatic NPC due to the tumor’s exceptional radiosensitivity and anatomical constraints limiting surgical accessibility.

In contemporary oncology practice, both intensity-modulated radiotherapy (IMRT) and volumetric modulated arc therapy (VMAT) constitute essential modalities for NPC management. How should a clinical decision be made on which of these techniques is required? In a dosimetric analysis by He et al. [4], VMAT achieved higher conformity indices and reduced mean doses to serial organs at risk relative to static IMRT, establishing its dual advantage in target coverage optimization and normal tissue protection for NPC. Nanda et al. [5] suggested that VMAT spared the cochlea and other organs at risk from high radiation doses, thereby reducing treatment-related toxicities and improving patients’ quality of life and survival outcomes. In the study by Guo et al. [6], VMAT achieved superior locoregional control compared while maintaining favorable toxicity burdens. Owing to its shortened treatment delivery time and dosimetric advantages—including superior target conformity and critical organ sparing—VMAT, an advanced form of radiation therapy, has increasingly supplanted IMRT as the preferred radiotherapy modality [7,8,9,10].

The geriatric nutritional risk index (GNRI), a pragmatic biomarker calculated from serum albumin concentration and body weight relative to ideal body mass, provides a clinically accessible composite measure reflecting integrated nutritional status and chronic inflammation. Grinstead et al. [11] revealed that nutritional vulnerability, captured by a low diagnostic GNRI and subsequent GNRI deterioration, significantly correlated with mortality risk in pancreatic cancer patients. In the study by Huang et al. [12], the GNRI was an independent prognostic factor for patients with head and neck cancer. Its prognostic utility has garnered substantial scientific interest across diverse solid tumors, with robust evidence establishing the GNRI as an independent predictor of survival outcomes also in other malignancies including clear cell renal cell carcinoma, esophageal adenocarcinoma, colorectal carcinoma, non-small-cell lung cancer, and prostate cancer [13,14,15,16,17].

Nevertheless, the specific relationship between the pretreatment GNRI and clinical prognosis in NPC populations remains insufficiently characterized, particularly within modern radiotherapy frameworks employing VMAT technology. To our knowledge, no prior investigations have comprehensively evaluated the GNRI’s prognostic significance exclusively in NPC cohorts treated with VMAT-based protocols. While the seminal work by Tang et al. [18] established the pretreatment GNRI as an independent prognostic determinant in NPC patients receiving IMRT-era chemoradiotherapy, the extrapolation of these findings to contemporary VMAT regimens remains problematic. Currently VMAT is broadly applied in the treatment of NPC; therefore, further investigation should be required to evaluate the impact of the GNRI for NPC patients under the condition of VMAT.

Hence, we intended to analyze the correlation between the GNRI and prognosis in NPC patients treated with VMAT in this study. Furthermore, we constructed a nomogram based on the GNRI to predict long-term prognosis of NPC patients who received VMAT and verified it.

## 2. Materials and Methods

### 2.1. Patients

Between January 2010 and November 2011, a total of 508 consecutive newly diagnosed NPC patients treated primarily with VMAT at our center were included in this retrospective study. The inclusion criteria were the following: (1) pathologically proven primary NPC; (2) newly diagnosed NPC patients without metastasis; (3) treatment with VMAT; (4) the availability of clinical and laboratory data; (5) the availability of image data. The exclusion criteria were as follows: (1) a second malignancy; (2) pregnancy or lactation; (3) metastatic disease at the time of diagnosis; (4) the receipt of conventional 2D/3D radiotherapy or IMRT; (5) severe medical complications; (6) incomplete treatment. Based on the criteria, 498 patients were included. The characteristics of patients are listed in Table 1. Patients were staged based on the 8th edition of the AJCC/UICC TNM staging system. This study was conducted in accordance with the Declaration of Helsinki and approved by the Institutional Review Board of Fujian Cancer Hospital (protocol code YKT2020-011-01, approved on 1 September 2020).

### 2.2. GNRI Measurement

In this study, the GNRI was calculated as follow: 1.487 × serum albumin concentration (g/L) + 41.7 × present body weight/ideal body weight (kg). The ideal body weight was computed according to the patient’s height and a body mass index (BMI) of 22 kg/m^2^ as follows: ideal body weight = 22 × square of height (m). This has been validated in previous studies [19,20]. The ratio of the patient’s actual weight to the ideal weight was set to 1 when the actual body weight surpassed the ideal weight. The optimal GNRI cut-off value was identified through receiver operating characteristic (ROC) curve analysis, subsequently enabling stratification of patients into a low-GNRI cohort and high-GNRI cohort.

### 2.3. Radiotherapy

Every patient in this study received definitive radiotherapy in the form of VMAT. The target volume and radiotherapy were applied in accordance with our treatment protocol previously described [21,22].

The total dose of the planning target volume was 69.7–70 Gy/31–35 fractions at 2–2.25 Gy/fraction for primary gross tumor volume or gross tumor volume of lymph nodes. While 59.5–62 Gy at 1.7–2 Gy/fraction was prescribed to the planning target volume of the high-risk region (CTV-1) and 52.7–56 Gy at 1.6–1.8 Gy/fraction was prescribed to planning the target volume of the low-risk region (CTV-2). The planning target volume was created based on each volume with an additional 3 mm margin, allowing for setup variability. A total of 151 patients with residual disease including a primary tumor and metastatic regional lymph nodes observed by imaging or nasopharyngoscope after radiotherapy received a boost of 4–12 Gy by VMAT.

### 2.4. Chemotherapy

Patients diagnosed with stage III-IV cancer underwent 2 to 4 cycles of cisplatin-based neoadjuvant chemotherapy, supplemented by 1 to 2 cycles of concurrent chemotherapy. Those with stage T1–2N1 disease were treated with 1 to 2 cycles of concurrent chemotherapy, while individuals at stage T1–2N0 were exclusively treated with radiation therapy. The neoadjuvant chemotherapy regimen consisted of either gemcitabine in combination with cisplatin or paclitaxel paired with cisplatin. Concurrent chemotherapy, which was based on cisplatin, was given concurrently with radiotherapy.

### 2.5. Follow-Up

Following the completion of radiotherapy, all patients were subject to a follow-up schedule consisting of evaluations every three months for the initial two years, followed by assessments every six months for the subsequent three years and then annually thereafter.

### 2.6. Statistical Methods

Statistical analysis was performed using SPSS statistical software version 22.0 (SPSS Inc., Chicago, IL, USA) and R version 3.4.0. An ROC curve analysis was conducted to determine the optimal cutoff point for the GNRI in predicting mortality. The area under the ROC curve (AUC) served as a metric to evaluate the prognostic significance of the GNRI. After then, the population of this study was randomly divided into a training cohort and a validation cohort according to a ratio of 7:3. The data from the training cohort were utilized to construct a predictive model for prognosis, while the data from the verification cohort were employed to internally validate the developed nomogram. The variables with *p* < 0.05 in the univariate analysis advanced to multivariate Cox regression analysis for further evaluation. The factors were considered to be independent prognostic indicators if they remained statistically significant in the multivariate Cox regression analysis (*p* < 0.05).

Employing the prognostic factors that we identified, we developed a nomogram designed to accurately predict the 3-year and 5-year overall survival (OS) rates. In order to ascertain the accuracy, differentiation, and clinical practicability of the nomogram, we performed a series of evaluations. This included calculating the concordance index (C-index), plotting the ROC curves, generating calibration curves, and conducting decision curve analysis (DCA). All *p*-values were calculated as two-tailed and statistical significance was defined as a *p* < 0.05.

## 3. Results

### 3.1. Treatment Outcomes

For the entire group, after a median follow-up period of 68 months (ranging from 4 to 110 months), we observed local or regional recurrence in 47 patients, distant metastasis in 81 patients, and a combined occurrence of distant metastasis with local or regional recurrence in 10 patients. The 5-year rates for locoregional failure-free survival, distant failure-free survival, disease-free survival, and overall survival were 90.6%, 83.7%, 71.5%, and 79.3%, respectively.

### 3.2. The Optimal Threshold for the GNRI

ROC curve analysis was performed to ascertain the optimal threshold for the GNRI. The optimal cut-off point of the GNRI was 102. The area under the ROC curve was 0.581 (95% CI, 0.518–0.644). Hence, we selected the cut-off point as 102 for survival analysis. Based on this value, patients were divided into two groups: the low-GNRI group (≤102) and the high-GNRI group (>102).

### 3.3. Patient Characteristics According to the Optimal Threshold for the GNRI

In the study, 348 patients were included in the training cohort and 150 patients were included in the validation cohort according to a ratio of 7:3. The baseline features of the two groups are summarized in Table 1. Importantly, there were no statistically significant differences in the baseline characteristics between the training and validation cohorts. According to the cut-off point, a total of 223 patients (44.8%) exhibited a high GNRI (>102), with 148 (42.5%) in the training cohort and 75 (50%) in the validation cohort.

### 3.4. Survival Analysis

**Figure 1:**
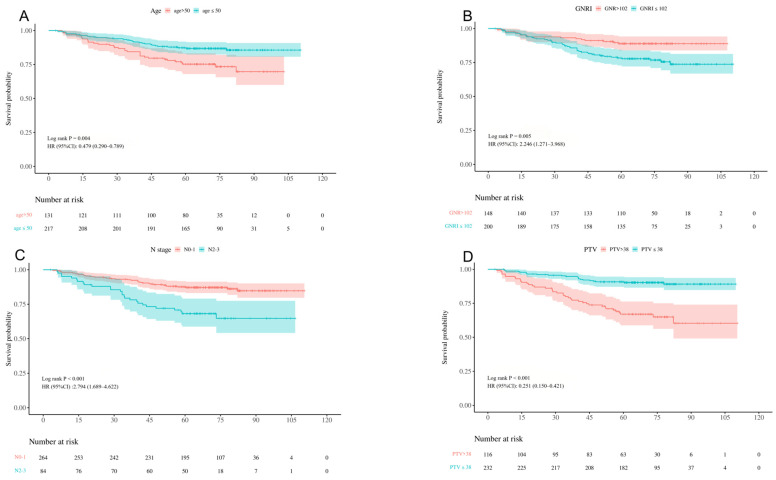
Kaplan–Meier curves for overall survival of NPC patients treated with VMAT stratified based on age (**A**), geriatric nutritional risk index (**B**), N-stage (**C**), and primary tumor volume (**D**).

The survival analysis incorporated a comprehensive set of variables, including age, gender, N-stage, T-stage, primary tumor volume (PTV), KPS score, boost, interruptions during radiotherapy, concurrent chemotherapy, and the GNRI. Univariate analysis indicated that the GNRI (*p* = 0.005), PTV (*p* < 0.001), age (*p* = 0.004), T-stage (*p* = 0.014) and N-stage (*p* < 0.001) were associated with the OS of NPC patients. Multivariate Cox regression analysis showed that the GNRI (*p* = 0.044), PTV (*p* < 0.001), age (*p* = 0.006) and N-stage (*p* < 0.001) were significantly correlated with the risk of death. The results of univariate analysis and multivariate analysis are shown in Table 2. The Kaplan–Meier method was used to build survival curves. Patients with a high GNRI had significantly better prognosis than other patients with a low GNRI (*p* = 0.004). Patients with younger age and lower N-stage and PTV also had higher survival rates (Figure 1).

### 3.5. Nomogram Construction

**Figure 2:**
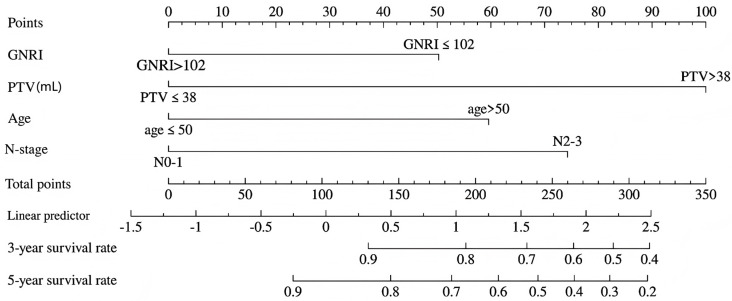
Prognostic nomogram for predicting 3-year and 5-year overall survival for NPC patients treated with VMAT.

**Figure 3:**
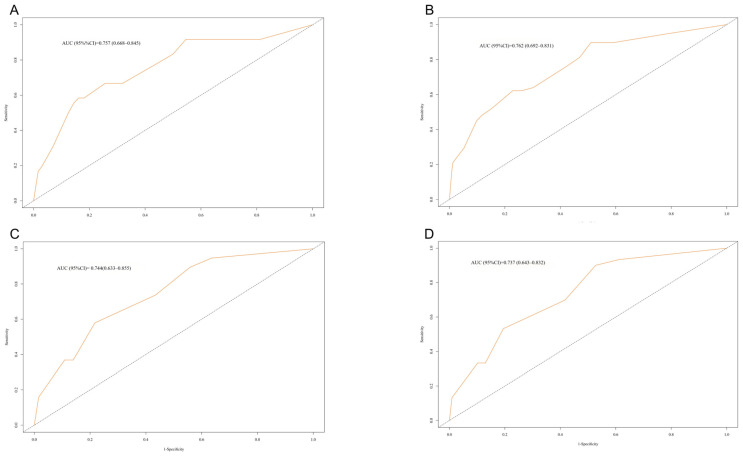
Receiver operating characteristic (ROC) curves for evaluating the nomogram for predicting 3-year (**A**) and 5-year (**B**) overall survival in the training cohort and 3-year (**C**) and 5-year (**D**) overall survival in the validation cohort.

Based on the significant independent prognostic factors identified in the training cohort, we developed a nomogram to predict 3- and 5-year OS for patients with nasopharyngeal carcinoma (Figure 2). The graphical representation demonstrates that higher total scores corresponded to diminished survival probabilities. The nomogram achieved a C-index of 0.757 (95% CI: 0.668–0.845) for 3-year OS prediction and a C-index of 0.762 (95% CI: 0.692–0.831) for 5-year OS prediction (Figure 3A,B). To implement the nomogram, clinicians should first locate each prognostic variable’s value on corresponding axes, sum the derived point scores, and then project this total vertically to predict 3- and 5-year mortality risk in NPC patients. Longer vertical projections on the total points scale indicate elevated mortality probabilities.

### 3.6. Internal Validation

**Figure 4:**
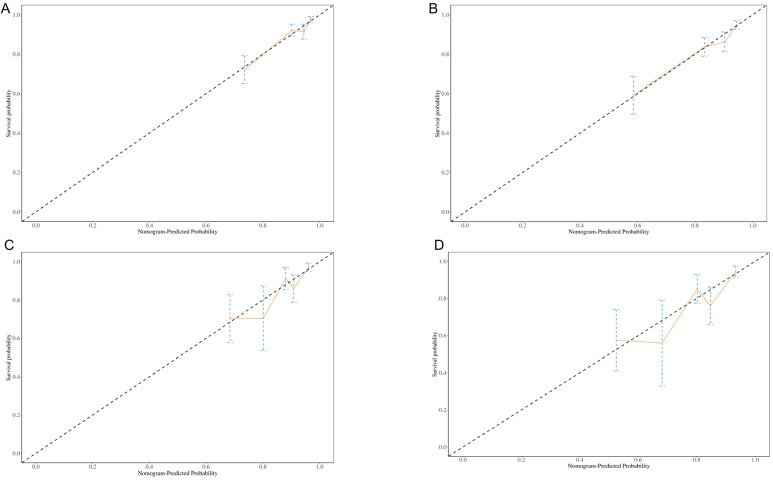
Calibration curves of the nomogram-predicted and actual measured survival probabilities at 3 years (**A**) and 5 years (**B**) for the training cohort and 3 years (**C**) and 5 years (**D**) for the validation cohort.

The predictive accuracy of the nomogram was considered clinically acceptable when the C-index exceeded 0.7 based on established criteria [23,24]. Our analysis demonstrated robust discrimination, with the nomogram achieving C-indices of 0.744 (95% CI: 0.633–0.855) and 0.737 (95% CI: 0.643–0.832) in the validation cohort for 3- and 5-year OS, respectively (Figure 3C,D). Calibration charts revealed optimal alignment with the 45-degree reference line for both 3-year and 5-year survival probabilities across cohorts. This indicated strong agreement between model-predicted outcomes and actual observations in both the training (Figure 4A,B) and validation cohorts (Figure 4C,D), with minimal deviations from ideal calibration.

**Figure 5:**
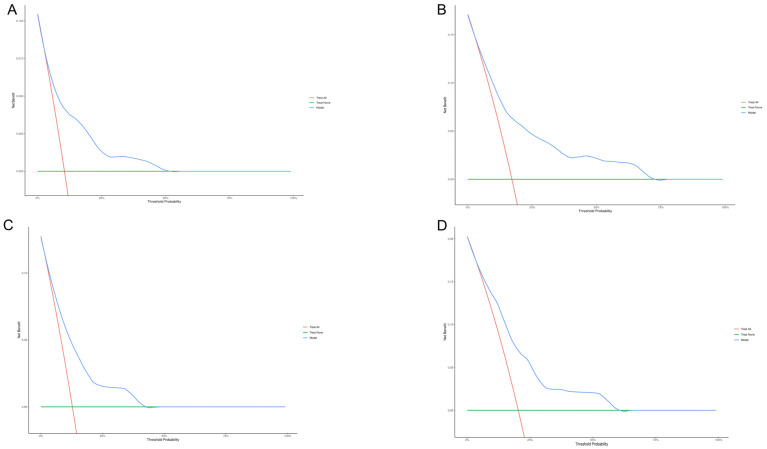
Decision curve analysis for the 3-year (**A**) and 5-year (**B**) survival of the training cohort and 3-year (**C**) and 5-year (**D**) survival of the validation cohort.

In addition, we performed decision curve analysis (DCA) to assess clinical utility of the nomogram in the two cohorts. The nomogram demonstrated superior benefit across threshold probability ranges, with great separation from the ‘treat-all’ and ‘treat-none’ reference curves indicating enhanced clinical utility for 3- and 5-year OS in the training cohort (Figure 5A,B) and validation cohort, respectively (Figure 5C,D).

## 4. Discussion

The advent of VMAT has revolutionized treatment strategies for NPC, with its widespread adoption in clinical practice. While previous studies on the prognostic significance of the GNRI have predominantly focused on IMRT, the prognostic implications of the GNRI under advanced VMAT protocols remain underexplored. Thus, we conducted this study to find the prognosis value of the GNRI and furthermore developed a nomogram model for predicting OS in VMAT-treated NPC patients. To our knowledge, this work is the first study to address the prognostic utility of ZGNRI in the VMAT era for NPC patients, with potential implications for adaptive radiotherapy protocol development. Our findings underscored the GNRI as an independent predictor of OS in this population, with a nomogram incorporating ZGNRI demonstrating robust discriminative ability and clinical utility. These results not only validate the prognostic relevance of nutritional status in NPC but also highlight the necessity of reevaluating traditional prognostic tools in the context of evolving radiotherapy modalities.

While many studies have revealed that IMRT improves nasopharyngeal carcinoma outcomes, prolonged treatment duration in IMRT may increase risks of positioning errors and secondary malignancies compared to those in VMAT [25,26,27,28]. A number of recent studies have shown that confirmed VMAT is a novel modality distinguished by its dynamic dose delivery, enhanced normal tissue sparing, and reduced treatment toxicity [7,8,9,29]. He et al. [4] conducted a study to compare dosimetric parameters and late toxicities between IMRT and VMAT among NPC patients. The maximum doses in the brainstem, spinal cord, temporal lobes, temporomandibular joint, optic chiasm, and lens were lower in VMAT than those in IMRT, where the median dose reduction ranged from 0.56 to 3.56 Gy (*p* < 0.05). VMAT could reduce ototoxicity, trismus, and temporal lobe injury. In conclusion, VMAT offers a number of advantages over IMRT in radiotherapy for NPC, including shorter treatment times, potentially reduced toxicity, and excellent target coverage with the sparing of critical structures. These advancements necessitate reevaluating the existing prognostic value of the GNRI in the VMAT era.

The GNRI, derived from serum albumin levels and body weight, has been widely used as a simple yet effective tool to assess nutritional status and predict survival outcomes in various cancer types. Huang et al. [12] conducted a meta-analysis to indicate that a low GNRI was significantly associated with poor OS in patients with head and neck cancer. Hirahara et al. [30] found that the GNRI was an important predictor of OS in an elderly population afflicted with gastric cancer. Prior studies on NPC, such as that by Tang et al. [18], established the GNRI as a prognostic marker in NPC patients treated with IMRT. However, the transition to VMAT introduces critical differences in dose delivery, toxicity profiles, and treatment efficiency, which may influence survival outcomes and confound the applicability of existing prognostic models. Our analysis bridges this gap, revealing that the GNRI retained its prognostic significance even under advanced VMAT protocols. The nomogram developed here, which integrated the GNRI with tumor volume, age, and N-stage, achieved C-indices exceeding 0.7 in both the training and validation cohorts, indicating strong predictive accuracy for 3- and 5-year OS. This tool provides clinicians with a practical, individualized risk stratification framework to guide therapeutic decision-making and patient counseling.

The association between a low GNRI and inferior survival aligns with evidence from other malignancies [14,15,16,17,30], reinforcing the role of malnutrition and systemic inflammation in cancer progression. Serum albumin, a component of the GNRI, reflects not only nutritional status but also chronic inflammation and immune dysfunction, both of which are implicated in tumor aggressiveness and treatment resistance [13,30]. Importantly, our cohort exhibited a GNRI cutoff of 102, slightly higher than thresholds reported in gastric or colorectal cancer studies [15,30], suggesting disease-specific variations in nutritional risk assessment. This underscores the need for the context-specific validation of the GNRI across cancer types and treatment modalities.

Several factors may contribute to the observed relationship between the GNRI and OS in NPC patients. First, malnutrition is common among NPC patients and is associated with impaired immune function, which can hinder the body’s ability to combat cancer cells and increase susceptibility to infections [31]. Malnutrition may further exacerbate the side effects of radiotherapy, such as mucositis and dysphagia, thereby affecting treatment tolerance and compliance [32,33]. Second, malnutrition is often accompanied by systemic inflammation, which has been shown to promote cancer progression and metastasis. The GNRI, by incorporating both nutritional status and inflammatory response, provides a comprehensive assessment of a patient’s overall health status.

Clinically, the integration of the GNRI into prognostic models holds significant implications for adaptive therapy strategies. For instance, patients with low GNRI scores may benefit from intensified nutritional support, closer surveillance, or adjunct therapies to mitigate treatment-related toxicities. Furthermore, the nomogram’s ability to quantify survival probabilities could facilitate risk-adapted trial designs, enabling the personalized allocation of novel therapies or dose-escalation regimens. The calibration and decision curve analyses further affirm the model’s clinical utility, as it outperforms “treat-all” or “treat-none” strategies across a wide range of threshold probabilities.

Nevertheless, several limitations warrant consideration. First, the retrospective design introduces potential selection bias, and unmeasured confounders such as socioeconomic factors or comorbidities may have influenced outcomes. Second, while internal validation demonstrated robustness, external validation in multicenter cohorts is essential to confirm generalizability. Finally, this study did not explore dynamic changes in the GNRI during or after treatment, which may offer additional prognostic insights. Future research should prioritize prospective validation of the nomogram in diverse populations, alongside investigations into the mechanistic links between the GNRI, immune modulation, and treatment response. Additionally, interventional studies evaluating whether nutritional optimization improves outcomes in low-GNRI patients are urgently needed.

## 5. Conclusions

In conclusion, this study establishes the GNRI as a pivotal prognostic marker in the VMAT era for NPC, offering a validated nomogram to refine survival prediction and therapeutic personalization. As radiotherapy paradigms continue to evolve, integrating nutritional metrics into prognostic frameworks will remain critical to advancing precision oncology.

However, this study is inherently limited by its retrospective, monocentric design, which may introduce selection bias and restrict generalizability. Additionally, this study lacks external multicenter validation. Future multi-institutional prospective studies should validate our nomogram while integrating complementary nutritional assessments.

## Figures and Tables

**Table 1:** Baseline characteristics of patients in the training and validation cohorts.

Variables		Training Cohort	Validation Cohort	*p*
Number		348	150	
Gender	male	269 (77.3%)	114 (76%)	0.752
	female	79 (22.7%)	36 (24%)	
Age	≤50 y	217 (62.4%)	106 (70.7%)	0.075
	>50 y	131 (37.6%)	44 (29.3%)	
N-stage	N0–1	264 (75.9%)	105 (70%)	0.171
	N2–3	84 (24.1%)	45 (30%)	
T-stage	T1–2	128 (36.8%)	60 (40%)	0.497
	T3–4	220 (63.2%)	90 (60%)	
KPS score	≤80	11 (3.2%)	8 (5.3%)	0.246
	90	337 (96.8%)	142 (94.7%)	
Con-CT	Yes	81 (23.3%)	41 (27.3%)	0.334
	No	267 (76.7%)	109 (72.7%)	
Int	Yes	26 (7.5%)	8 (5.3%)	0.385
	No	322 (92.5%)	142 (94.7%)	
Boost	Yes	97 (27.9%)	53 (35.3%)	0.096
	No	251 (72.1%)	97 (64.7%)	
GNRI	≤102	200 (57.5%)	75 (50%)	0.124
	>102	148 (42.5%)	75 (50%)	
Alb (g/L)	≤40	147 (42.2%)	59 (39.3%)	0.545
	>40	201 (57.8%)	91 (60.7%)	
BMI (Kg/m^2^)	≤22	148 (42.5%)	64 (42.7%)	0.977
	>22	200 (57.5%)	86 (57.3%)	

Con-CT, concurrent chemotherapy; Int, interruption during radiotherapy; GNRI, geriatric nutritional risk index; Alb, albumin; BMI, body mass index.

**Table 2:** Univariate and multivariate analysis of predictive factors for the patients with NPC.

Variables	Univariate Analysis		Multivariate Analysis	
	HR (95% CI)	*p*	HR (95% CI)	*p*
GNRI				
GNRI > 102	1.00 (Reference)		1.00 (Reference)	
GNRI ≤ 102	2.24 (1.27–3.96)	0.005	1.81 (1.02–3.22)	0.044
PTV				
PTV > 38 mL	1.00 (Reference)		1.00 (Reference)	
PTV ≤ 38 mL	0.25 (0.15–0.42)	<0.001	0.31 (0.18–0.52)	<0.001
Age				
age > 50	1.00 (Reference)		1.00 (Reference)	
age ≤ 50	0.48 (0.29–0.79)	0.004	0.50 (0.30–0.82)	0.006
Sex				
Female	1.00 (Reference)			
Male	1.83 (0.90–3.72)	0.093		
T-stage				
T1–2	1.00 (Reference)		1.00 (Reference)	
T3–4	2.11 (1.16–3.82)	0.014	1.40 (0.48–2.51)	0.812
N-stage				
N0–1	1.00 (Reference)		1.00 (Reference)	
N2–3	2.79 (1.69–4.62)	<0.001	2.40 (1.44–4.00)	<0.001
KPS				
KPS 90	1.00 (Reference)			
KPS ≤ 80	1.50 (0.47–4.79)	0.493		
Con CT				
No	1.00 (Reference)			
Yes	0.88 (0.48–1.63)	0.688		
Int				
Int < 5	1.00 (Reference)			
Int ≥ 5	0.85 (0.31–2.34)	0.752		
Boost				
No	1.00 (Reference)			
Yes	1.30 (0.77–2.19)	0.333		

GNRI, geriatric nutritional risk index; PTV, primary tumor volume; Con-CT, concurrent chemotherapy; Int, interruption during radiotherapy; HR, hazard ratio; CI, confidence interval.

## Data Availability

The data that support the findings of this study are available from the corresponding author upon reasonable request.

## Abbreviations

The following abbreviations are used in this manuscript:

GNRI	Geriatric nutritional risk index
VMAT	Volumetric modulated arc therapy
PTV	Primary tumor volume
NPC	Nasopharyngeal carcinoma
ROC	Receiver operating characteristic
OS	Overall survival
C-index	Concordance index
DCA	Decision curve analysis
Alb	Albumin
BMI	Body mass index
